# Progression characteristics of biliary atresia during the waiting period for liver transplantation: a single-center observational study

**DOI:** 10.3389/fmed.2025.1679733

**Published:** 2025-10-24

**Authors:** Chenyu Yang, Jiqiang Hu, Bingqian Tan, Qiang Xiong, Ruijue Wang, Jianyang Hu, Ying Le, Xiaoke Dai, Mingman Zhang

**Affiliations:** Department of Hepatobiliary Surgery Children’s Hospital of Chongqing Medical University, National Clinical Research Center for Child Health and Disorders, Chongqing Key Laboratory of Structural Birth Defect and Reconstruction, Chongqing Municipal Health Commission Key Laboratory of Children’s Vital Organ Development and Diseases, Chongqing, China

**Keywords:** biliary atresia, intraoperative cholangiogram, liver cirrhosis, natural progression, Kasai portoenterostomy (KPE)

## Abstract

**Background:**

Biliary atresia (BA) is a progressive, fibro-obliterative cholangiopathy of infancy that inevitably leads to cirrhosis and liver failure without intervention. While the rapid progression of BA is widely recognized, the precise dynamics of its natural history remain poorly quantified due to the confounding effects of surgical intervention.

**Aims:**

This study aimed to characterize the natural progression of BA cirrhosis by analyzing the trajectory of clinical, laboratory, and histological changes during the unique window between diagnostic confirmation by intraoperative cholangiography and subsequent liver transplantation in children who did not undergo Kasai portoenterostomy.

**Methods:**

In this single-center, observational study, we analyzed 153 children with cholangiography-confirmed BA who underwent living donor LT without primary Kasai portoenterostomy. The pathological progression of cholestatic cirrhosis and laboratory parameters such as hematological examination and liver function in children with hepatic cholestatic cirrhosis at the two time points were compared to find factors that can predict the progression of BA cirrhosis.

**Results:**

The cohort comprised 153 children with cholangiography-confirmed BA who underwent liver transplantation. The mean age at diagnosing BA was 78.64 ± 31.43 days. The mean interval from exploration to liver transplantation (disease progression time) was 83.36 ± 28.37 days. Within this short period, 77.12% (118/153) of children progressed to end-stage (Stage IV) cirrhosis. Longitudinal analysis revealed a significant increase in white blood cells (WBC, 3.09 ± 5.31 × 10^9^/L), neutrophil percentage (NE%, 7.42 ± 13.77%), total bilirubin (TBIL, 121.9 ± 99.51 μmol/L), and serum ammonia (SA, 6.97 ± 25.59 μmol/L), alongside a significant decrease in hemoglobin (HB, −4.7 ± 15.83 g/L), platelets (PLT, −148.50 ± 154.40 × 10^9^/L), and albumin (ALB, −4.60 ± 7.28 g/L) (all *p* < 0.05). A predictive model incorporating initial fibrosis stage, waiting time, and changes in NE% and SA demonstrated good accuracy (75.96% in training set, 72.00% in validation set) for forecasting cirrhosis progression.

**Conclusion:**

This study provides unprecedented quantification of the rapid natural progression of BA cirrhosis, revealing a critical 3 month window of clinical deterioration. The dynamic changes in routinely available laboratory markers, particularly neutrophil percentage and ammonia, offer clinically accessible indicators for risk stratification and monitoring, forming a practical “dashboard” for managing BA patients awaiting transplantation in resource-varied settings.

## 1 Introduction

Biliary atresia (BA) is a severe hepatobiliary disease primarily occurring in neonates. It is characterized by the obstruction of both intrahepatic and extrahepatic bile ducts, which prevents the normal drainage of bile, leading to its accumulation in the liver. This subsequently causes liver damage, cirrhosis, and even liver failure (1–3). The exact cause of BA remains unclear, but current research supports a multifactorial hypothesis. It suggests that genetic factors, viral infections (such as cytomegalovirus and rotavirus), exposure to toxic chemicals, and autoimmune dysregulation may be associated with the onset and progression of BA. Geographical and racial differences are particularly evident in the incidence of BA. In Asia, the overall incidence of BA is approximately 1.06 to 1.8 per 10,000 live births (4, 5). In China, BA has been classified as a rare disease. However, the incidence of BA is significantly lower in Europe and the United States, with only 0.52 to 0.66 cases per 10,000 live births (6–8). This difference may be due to variations in genetic backgrounds, environmental factors, or the interaction between the two.

Kasai portoenterostomy (KPE) procedure is the primary and classic treatment for BA (1, 2). For children with BA who have been diagnosed and treated early, this surgery can significantly improve outcomes. Without timely treatment, persistent bile accumulation leads to progressive liver cirrhosis, ultimately resulting in death by the 2 years old (1). However, not all BA patients respond effectively to KPE. For this group of patients, liver transplantation becomes a life-saving option. Although liver transplantation is an effective treatment, it comes with a range of challenges, including surgical risks, long-term immunosuppressive therapy, and potential mid- to long-term complications.

Therefore, early identification of the cause of neonatal jaundice is a necessary and sufficient condition for determining the prognosis of children with BA. This study retrospectively analyzed the biochemical data of the natural progression of the disease in BA patients, and explored the dynamic changes in laboratory test indicators during the progression of BA and methods for predicting the progression of cirrhosis in BA patients. This analysis aims to help early identification of liver pathological changes in BA and determine the appropriate timing of liver transplantation.

## 2 Materials and methods

### 2.1 Study subjects and inclusion/exclusion criteria

This study included children diagnosed with BA who were admitted to the Department of Hepatobiliary Surgery at Children’s Hospital of Chongqing Medical University between January 1, 2018, and June 30, 2023.

Inclusion criteria: (1) Children diagnosed with BA via “intraoperative cholangiogram” at our center who subsequently underwent living donor liver transplantation; (2) Willingness of a relative to donate liver tissue meeting transplant requirements; (3) Approval of the liver transplantation treatment plan by the hospital ethics committee and the Chongqing Red Cross Society; (4) Written informed consent provided by the legal guardian (s) of the recipient and the donor or their relatives.

Exclusion criteria: (1) BA children with contraindications for pediatric liver transplant surgery; (2) Children who underwent Kasai portoenterostomy after BA diagnosis via “intraoperative cholangiogram” or did not receive liver transplantation at our center; (3) Children diagnosed with BA at other medical institutions who received liver transplantation in our department; (4) Children who, for various reasons, did not complete the liver transplantation procedure; (5) Children with incomplete key clinical data required for this study (e.g., postoperative pathological records); (6) Request by the legal guardian (s) of the recipient, the donor, or their relatives to withdraw from the study.

The research protocol was approved by the Medical Research Ethics Committee of our institution, and written informed consent was obtained from the research participants or their legal guardians. The research process strictly adhered to the principles outlined in the Declaration of Helsinki.

### 2.2 Clinical data collection

Clinical data for children with BA who underwent both “intraoperative cholangiogram” and “liver transplantation” at Chongqing Medical University Affiliated Children’s Hospital were retrieved from the hospital’s Clinical Big Data Center. The following data were collected:

(1) General data: Patient ID, gender, age, date of intraoperative cholangiogram, date of liver transplantation, and progression time. (2) Laboratory indicators: (1) Hematological analysis: Red blood cell (RBC), white blood cell (WBC), hemoglobin (Hb), platelet (PLT) count, neutrophil (NE) count and percentage, lymphocyte (LYM) count and percentage, peripheral blood mononuclear cell (PBMC) count and percentage, eosinophil (E) count and percentage. (2) Liver function: Total bilirubin (TBIL), direct bilirubin (DBIL), indirect bilirubin (IBIL), aspartate aminotransferase (AST), alanine aminotransferase (ALT), gamma-glutamyl transferase (GGT), albumin (ALB), globulin (GLB), total bile acid (TBA), total protein (TP), prealbumin (PA). (3) Other indicators: Serum ammonia (SA), serum lactate (LAC), serum creatinine (Cr). All laboratory indicators were recorded at the time of intraoperative cholangiogram and liver transplantation, specifically preoperative data obtained during the same hospital admission for surgery. The lower limit for serum creatinine (Cr) detection in our center is 13.3 μmol/L. Values below this threshold were clinically reported as “<13.3 μmol/L,” and in this study, these values were recorded as 13.3 μmol/L for analysis. (3) Pathological diagnosis of the diseased liver obtained from intraoperative cholangiogram and liver transplantation in recipients. The pathological evaluation of liver fibrosis in this study was performed according to the Batts–Ludwig scoring system (9, 10), a well-validated and widely accepted histopathological framework for staging chronic liver disease. This system classifies fibrosis into the following stages: Stage I: Portal inflammation with non-purulent cholangitis, without fibrosis; Stage II: Bile duct proliferation with periportal or portal fibrosis; Stage III: Bridging fibrosis (fibrous septa extending between portal tracts); Stage IV: Established cirrhosis.

For data not recorded in the big data center or missing data, manual searches in the electronic medical records system were performed to complete the information. Missing values that were insufficient to meet exclusion criteria were replaced with “NA” and saved as raw data for subsequent analysis.

### 2.3 Statistical analysis

Continuous variables that follow a normal distribution were described using “mean ± standard deviation (x̅ ± SD),” while continuous variables that do not follow a normal distribution were described using “median [interquartile range] (M [Q1, Q3]).” Categorical variables were described using “frequency (percentage).” Outliers were defined as values with absolute values greater than |x̅ ± 3SD|. Missing values were imputed using the mean imputation method.

Data were analyzed and visualized using R (version 4.2.2) and GraphPad Prism 9. The R packages employed included MASS, effects, brant, and corrplot, among others. For continuous variables following a normal distribution, intergroup differences were analyzed using *t*-tests. Specifically, for data obtained at the time of intraoperative cholangiogram and liver transplantation—where disease progression was the sole factor under consideration—the paired nature of the measurements justified the use of paired *t*-tests to compare these two time points. For continuous variables not conforming to a normal distribution, non-parametric tests (Mann–Whitney *U* test) were used to evaluate intergroup differences. Ordinal logistic regression was employed to identify factors influencing liver pathological progression in BA patients, and relevant predictive models were constructed and validated. A significance level of *α* = 0.05 was set, with *p* < 0.05 considered statistically significant.

## 3 Result

### 3.1 Most children show obvious liver fibrosis when they were diagnosed with BA

In this study, 153 children were diagnosed with BA through “intraoperative cholangiogram.” Among them, 81 were male and 72 were female. The average age at the time of exploration was 78.64 ± 31.43 days. Pathological results of liver biopsy during intraoperative cholangiography were evaluated according to the Batts–Ludwig scoring system. The distribution of liver fibrosis stages was as follows: Stage II in 6 children (3.92%), Stage II–III in 55 children (35.95%), Stage III in 48 children (31.37%), Stage III–IV in 22 children (14.38%), and Stage IV in 22 children (14.38%). The distribution of liver fibrosis stages at intraoperative cholangiogram demonstrated that the majority of patients (81.7%, 125/153) already exhibited significant fibrosis or cirrhosis (Stage III or higher) at time of definitive diagnosis. The high detection rate of fibrosis or cirrhosis at the time of definitive diagnosis highlights the early rapid progression of BA cirrhosis. The detailed data can be seen in Table 1.

**Table 1:** Clinical data at the time of biliary exploration in a child with BA.

Project		Overall condition (*n* = 153)
Sex	Boys	81 (52.94%)
	Girls	72 (47.06%)
Age at the time of intraoperative cholangiogram (days)		78.64 ± 31.43
Stages of cholestatic cirrhosis	II	6 (3.92%)
	II–III	55 (35.95%)
	III	48 (31.37%)
	III–IV	22 (14.38%)
	IV	22 (14.38%)
Hematological analysis	RBC (×10^9^/L)	3.57 ± 0.48
	WBC (×10^9^/L)	12.06 ± 3.24
	PLT (×10^9^/L)	450.04 ± 144.54
	HB (g/L)	106.41 ± 14.13
	NE (×10^9^/L)	3.11 ± 1.17
	LYM (×10^9^/L)	7.91 ± 2.55
	PBMC (×10^9^/L)	0.57 ± 0.36
	E (×10^9^/L)	0.47 ± 0.27
	NE%	25.96 ± 8.52
	LYM%	65.11 ± 8.71
	E%	3.98 ± 2.01
	PBMC%	4.68 ± 1.89
Liver function	TBIL (μmol/L)	174.31 ± 45.82
	DBIL (μmol/L)	130.74 ± 42.56
	IBIL (μmol/L)	35.61 ± 17.66
	AST (U/L)	264.49 ± 159.61
	ALT (U/L)	178.34 ± 116.71
	AST/ALT	1.65 ± 0.71
	GGT (U/L)	430 [215, 710]
	TBA (μmol/L)	151.54 ± 63.22
	TP (g/L)	58.07 ± 6.00
	ALB (g/L)	41.60 ± 4.03
	GLB (g/L)	16.47 ± 5.13
	PA (g/L)	113.99 ± 39.76
Cr (μmol/L)		20.12 ± 6.31
LAC (mmol/L)		3.93 ± 1.72
SA (μmol/L)		49.18 ± 23.11

### 3.2 Liver fibrosis progresses rapidly to end-stage cirrhosis pre-transplant

Of the 153 children diagnosed with biliary atresia via intraoperative cholangiography, the mean waiting period until liver transplantation was 83.36 ± 28.37 days. The mean age at the time of liver transplantation was 162 ± 29.49 days. The liver biopsy pathological results at the time of liver transplantation were assessed according to the criteria for cholestatic cirrhosis. Pathological assessment of explanted livers using the Batts–Ludwig scoring system for cholestatic cirrhosis revealed the following distribution of fibrosis stages at transplantation: Stage II in 1 child (0.65%), Stage II–III in 1 child (0.65%), Stage III in 19 children (12.42%), Stage III–IV in 14 children (9.15%), and Stage IV in 118 children (77.12%).

Of the entire cohort, 35 children (22.88%) did not experience cirrhosis progression, including the 22 children who already had Stage IV cirrhosis at the time of exploration. Additionally, 36 children (22.53%) had progression of 0.5 stages, 39 children (25.49%) progressed by 1 stage, 38 children (24.83%) progressed by 1.5 stages, and 5 children (3.27%) progressed by 2 stages. In total, 96 children (62.74%) progressed to Stage IV cirrhosis. Detailed data are shown in Table 2.

**Table 2:** Clinical data at the time of liver transplantation in a child with BA.

Projects		Overall condition (*n* = 153)
Age at the time of LT (d)		162 ± 29.49
Progression time (d)		83.36 ± 28.37
Stages of cholestatic cirrhosis	II	1 (0.65%)
	II–III	1 (0.65%)
	III	19 (12.42%)
	III–IV	14 (9.15%)
	IV	118 (77.12%)
Progression of cholestatic cirrhosis in BA children	No progression	35 (22.88%)
	0.5 stage	36 (23.53%)
	1 stage	39 (25.49%)
	1.5 stage	38 (24.83%)
	2 stage	5 (3.27%)
Hematological analysis	RBC (×10^9^/L)	3.65 ± 0.45
	WBC (×10^9^/L)	15.25 ± 5.68
	PLT (×10^9^/L)	294.86 ± 132.81
	HB (g/L)	103.10 ± 29.27
	NE (×10^9^/L)	5.04 ± 3.43
	LYM (×10^9^/L)	9.03 ± 3.78
	PBMC (×10^9^/L)	0.65 ± 0.93
	E (×10^9^/L)	0.62 ± 2.13
	NE%	33.38 ± 13.24
	LYM%	58.36 ± 13.56
	E%	2.66 ± 2.38
	PBMC%	4.39 ± 1.48
Liver function	TBIL (μmol/L)	296.32 ± 102.30
	DBIL (μmol/L)	217.36 ± 89.26
	IBIL (μmol/L)	56.94 ± 33.40
	AST (U/L)	461.34 ± 241.35
	ALT (U/L)	261.29 ± 135.44
	AST/ALT	1.96 ± 0.96
	GGT (U/L)	414 [200, 734]
	TBA (μmol/L)	258.88 ± 104.80
	TP (g/L)	62.54 ± 8.32
	ALB (g/L)	37.09 ± 6.86
	GLB (g/L)	25.57 ± 8.64
	PA (g/L)	79.06 ± 59.16
Cr (μmol/L)		16.52 ± 4.17
LAC (mmol/L)		3.34 ± 1.43
SA (μmol/L)		55.12 ± 19.91

### 3.3 BA progression is associated with significant shifts in peripheral immune cell profiles

The above data clearly reflect the rapid progression of cirrhosis in the liver of children with BA. Within approximately 3 months, the proportion of children with Stage IV cirrhosis increased by 62.74%. This provides clinical evidence for the necessity of early surgical intervention in BA patients to delay liver fibrosis progression and alleviate liver function damage. Based on this, the study used paired *t*-tests to explore changes in peripheral blood cell composition during disease progression in BA children. The study revealed trends in the changes of various blood parameters, such as RBC, WBC, PLT, HB, NE, LYM, as well as E and PBMC, as liver fibrosis worsened.

**Figure 1:**
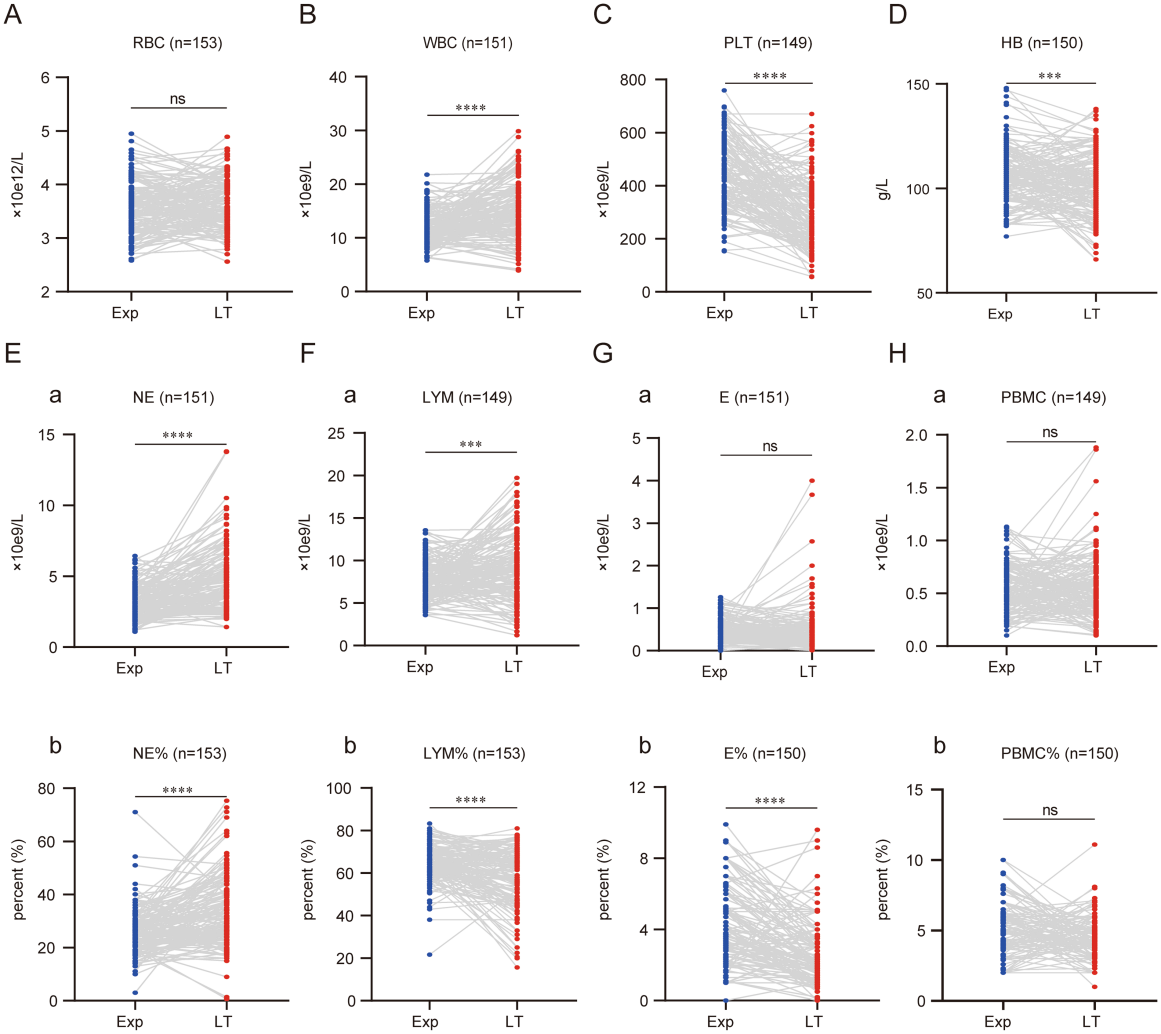
Cellular composition of peripheral blood of children with BA changes significantly with disease progression. Graphs A to H display comparisons between experimental (Exp) and long-term (LT) groups for various blood parameters. Each graph shows individual data points connected by lines between the two groups, with statistical significance indicated above each. Parameters include RBC, WBC, PLT, HB, NE, LYM, E, and PBMC, with respective units and sample sizes. (ns represents *p* > 0.05, * represents *p* < 0.05, ** represents *p* < 0.01, *** represents *p* < 0.001, **** represents *p* < 0.0001).

This study found that with the progression of BA and the worsening of liver fibrosis, the RBC count did not show a significant decrease in the affected children. However, BA children often present with anemia, as evidenced by 132 children (86.27%) having HB levels <120 g/L at the time of exploration, and 131 children (85.62%) at the time of liver transplantation. Although the number and proportion did not change significantly, comparison before and after showed that the mean HB decreased by 4.7 ± 15.83 g/L at the time of liver transplantation, indicating that as cirrhosis worsened, the anemia status also became more severe (Figures 1A,D).

WBC levels increased significantly with the progression of cirrhosis, with the mean WBC at the time of liver transplantation rising by (3.09 ± 5.31) × 10^9^/L compared to before. In contrast, PLT showed a marked decrease, with the mean PLT at liver transplantation dropping by (148.50 ± 154.40) × 10^9^/L compared to before (Figures 1B,C).

By analyzing NE and LYM, two blood cells that undergo notable changes during children’s growth and development, it was found that the NE count and percentage both increased significantly as the disease progressed. The NE count increased by (1.66 ± 2.14) × 10^9^/L, and the NE percentage increased by (7.42 ± 13.77)% compared to the time of exploration (Figures 1Ea,b). In contrast, LYM showed an opposite trend in terms of count and percentage. As the disease progressed, the LYM count at the time of liver transplantation increased significantly by (1.25 ± 4.14) × 10^9^/L, while the LYM percentage decreased significantly by (6.75 ± 14.68)% compared to before (Figures 1Fa,b). The absolute values of E and PBMC did not show significant changes. However, the percentage of E decreased with disease progression, showing a reduction of (1.43 ± 2.07)% compared to before (Figures 1G,H,a,b).

### 3.4 Progressive hepatic dysfunction in BA: worsening cholestasis and declining synthetic function

**Figure 2:**
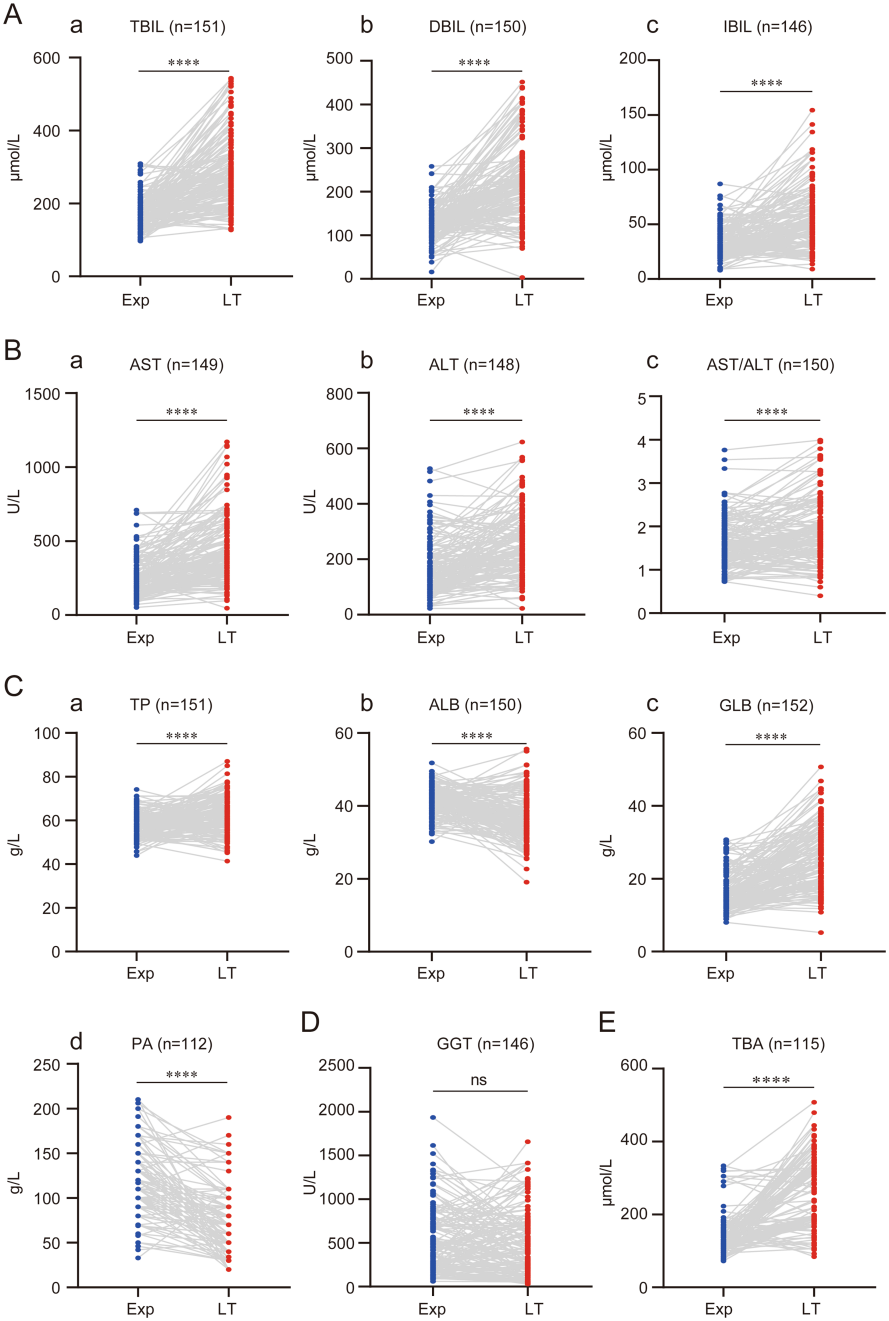
Progressive regression of liver function with disease progression in children with BA. **(A)** Progressive elevation of bilirubin in children with BA. **(B)** Progressive elevation of transaminases in children with BA. **(C)** Synthesis dysfunction of the liver in children with BA. **(D)** Changes in GGT in children with BA. **(E)** Changes in TBA in children with BA. (ns represents *p* > 0.05, * represents *p* < 0.05, ** represents *p* < 0.01, *** represents *p* < 0.001, **** represents *p* < 0.0001).

The liver dysfunction in children with BA may even lead to liver failure. All 153 children in this study had varying degrees of liver dysfunction at the time of exploration. Therefore, we investigated the changes in liver function as liver cirrhosis progressed. This study found that jaundice in BA children progressively worsened. At the time of liver transplantation, TBIL, DBIL, and IBIL increased by an average of (121.9 ± 99.51) μmol/L, (87.44 ± 91.68) μmol/L, and (20.83 ± 28.22) μmol/L, respectively, compared to the time of exploration (Figure 2A). AST and ALT, the two main enzymes indicating liver damage, also showed a progressive increase. AST and ALT increased by an average of (199.7 ± 212.3) U/L and (79.26 ± 115.1) U/L, respectively, with AST showing a more significant increase. Consequently, the AST/ALT ratio also increased, with an average increase of 0.266 ± 0.59 compared to before (Figure 2B).

TP, ALB, and GLB reflect liver synthetic function. As the disease progressed, the liver’s synthetic capacity in BA children also declined. However, the study found that using <60 g/L as the cutoff for reduced TP, 99 children (64.71%) had reduced TP at the time of exploration, while only 54 children (35.29%) had reduced TP at the time of liver transplantation. This suggests that TP actually increased with disease progression, with an average increase of (4.70 ± 9.62) g/L compared to before (Figures 2C,a). Further analysis of ALB and GLB levels revealed that the increase in TP was mainly due to the increase in GLB, while ALB significantly decreased. ALB decreased by an average of (4.60 ± 7.28) g/L, while GLB increased by (9.22 ± 8.70) g/L compared to before (Figures 2Cb,c).

The decrease in PA further confirmed that as the disease progressed, the liver’s synthetic function in BA children worsened. At the time of liver transplantation, PA decreased by an average of (43.01 ± 42.66) g/L compared to before (Figures 2C,d). GGT, one of the key markers of bile duct injury and an important reference for diagnosing BA, did not show significant changes between the time of exploration and liver transplantation. This suggests that GGT might not serve as a reliable marker for evaluating BA disease progression (Figure 2D). This study also found that TBA progressively increased, with an average increase of (111.10 ± 108.70) μmol/L compared to before (Figure 2E).

**Figure 3:**
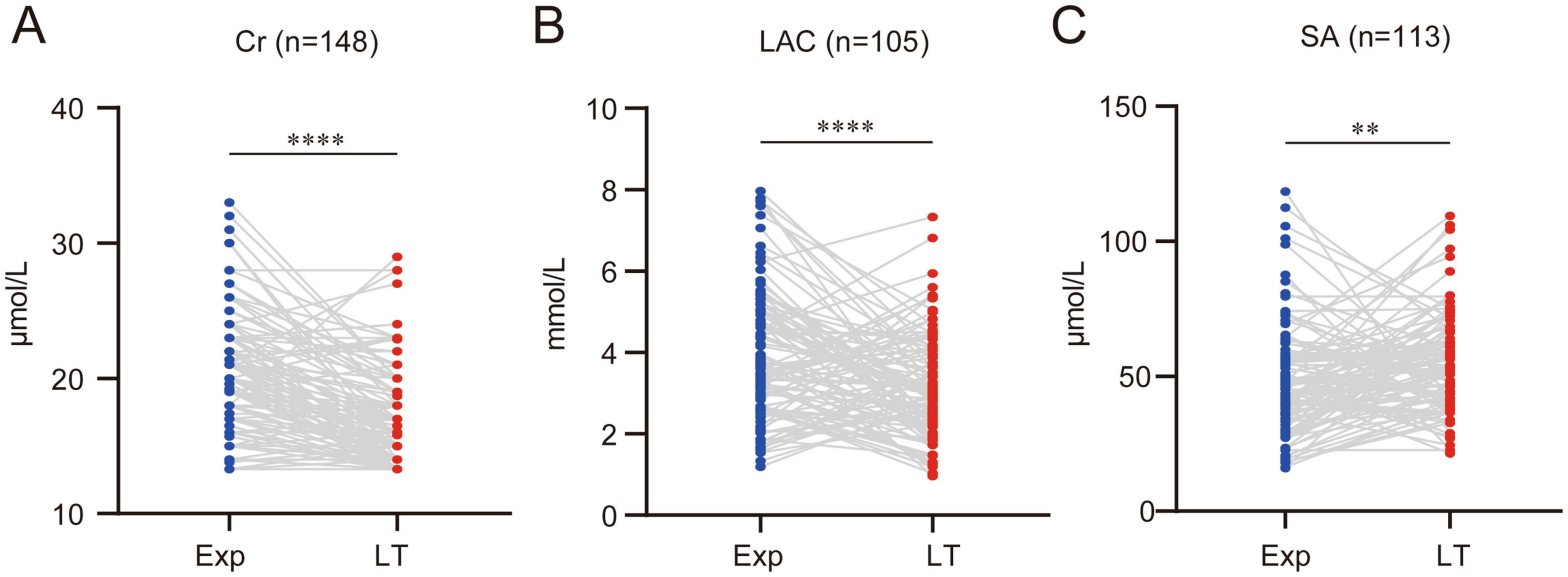
With the decline of liver function, several other biochemical indicators also change accordingly. **(A)** Serum creatinine (Cr) levels significantly decreased. **(B)** Serum lactate (LAC) levels significantly declined. **(C)** Serum ammonia (SA) levels significantly increased. (ns represents *p* > 0.05, * represents *p* < 0.05, ** represents *p* < 0.01, *** represents *p* < 0.001, **** represents *p* < 0.0001).

Additionally, some liver function-related markers were evaluated. Cr and LAC levels significantly decreased at the time of liver transplantation compared to before, with average reductions of (3.58 ± 3.87) μmol/L and (0.77 ± 1.91) mmol/L, respectively (Figures 3A,B). SA increased significantly due to progressive liver cirrhosis, with an average increase of (6.97 ± 25.59) μmol/L compared to before (Figure 3C).

### 3.5 Constructing a model to predict the progression of liver cirrhosis in BA

Given that several laboratory indicators undergo significant changes as BA progresses, this study aims to construct a model that can predict the progression of liver cirrhosis. The model will help monitor disease progression in BA children waiting for liver transplantation and assist in determining the appropriate timing for liver transplant. In this study, the pathological changes of the BA-affected liver at the time of liver transplantation were used as the outcome variable, and ordered logistic regression was used to select predictive indicators. Since 22 patients (14.38%) were already at cirrhosis Stage IV at the time of the exploration, marking the endpoint of the observation outcome, these patients were excluded from the model construction to reduce their influence. Additionally, due to the presence of missing values for different variables in individual patients, deleting all individuals with missing data would significantly reduce the number of patients eligible for inclusion in the model. Therefore, this study used mean imputation for missing values. Furthermore, because ordered logistic regression requires at least 8 individuals for each outcome category, 1 individual each from liver cirrhosis Stage II and Stage II–III at the time of liver transplantation was excluded from the model construction.

**Figure 4:**
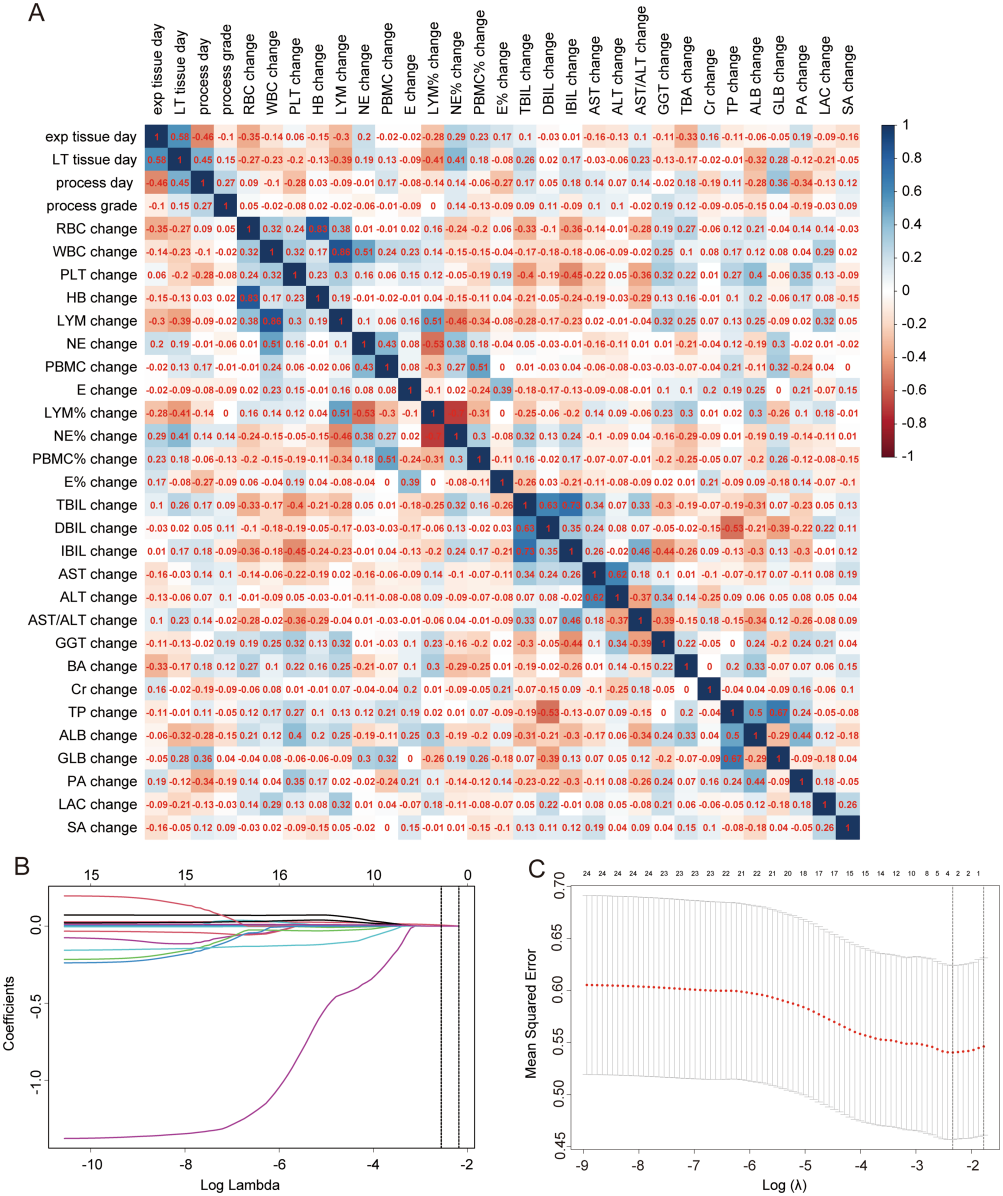
Collinearity analysis and LASSO regression. **(A)** Collinearity analysis for continuous variables, with numbers in squares representing correlation coefficients between two continuous variables. **(B)** LASSO coefficient path diagram. **(C)** Cross-validation curve for LASSO regression analysis.

The model construction included various factors such as the age at the time of exploration (exp tissue day), age at liver transplantation (LT tissue day), progression time (process day), liver cirrhosis staging at the time of exploration (exp result), liver cirrhosis staging at liver transplantation (LT result), and differences in laboratory indicators such as RBC, WBC, PLT, HB, NE, LYM, PBMC, E, NE%, LYM%, E%, PBMC%, TBIL, DBIL, IBIL, AST, ALT, AST/ALT, GGT, TBA, TP, ALB, GLB, PA, Cr, LAC, and SA between the exploration and liver transplantation periods. Collinearity analysis was conducted on continuous variables (see Figure 4A), and some indicators were found to exhibit collinearity. Therefore, LASSO regression was employed to select factors that could be used in the ordered logistic regression model. When the minimum standard error of *λ* was 0.1692944, progression time, NE% change, ALB change, and SA change were selected as independent variables (Figures 4B,C). These variables were then combined with the “exp result” to construct an ordered logistic regression model with “LT result” as the dependent variable (Tables 3, 4). The dataset was divided into a validation set and a training set in an 8:2 ratio for validation. The accuracy of the training set was 75.96%, with an error rate of 24.04%, while the accuracy of the validation set was 72.00%, with an error rate of 28.00% (Table 5).

**Table 3:** Parameters of independent variables in the ordinal logistic regression model for the progression of cholestatic liver cirrhosis in children with BA.

Project	Regression coefficient	SD	*t*-value	*p*-value
Exp result L	−8.05	0.36	−22.27	6.65 × 10^−110^
Exp result Q	7.67	0.48	15.94	3.39 × 10^−57^
Exp result C	−2.58	0.34	−7.62	2.54 × 10^−14^
Process day	0.02	0.01	2.47	0.01
NE% change	0.03	0.02	1.79	0.07
ALB change	−0.03	0.03	−1.04	0.30
SA change	0.01	0.01	1.17	0.24

“exp result L” represents the linear term; “exp result Q” represents the quadratic term; “exp result C” represents the cubic term.

**Table 4:** Thresholds of the dependent variable in the ordinal logistic regression model.

LT result	Cutoff	SD	*t*-value	*p*-value
III Stage/III–IV Stage	−3.28	0.63	−5.22	1.82 × 10^−7^
III–IV Stage/IV Stage	−2.48	0.633	−3.92	8.78 × 10^−5^

**Table 5:** Validation of the ordinal logistic regression model.

Predict situation	Training set actual situation			Validation set actual situation		
	III Stage	III–IV Stage	IV Stage	III Stage	III–IV Stage	IV Stage
III Stage	2	2	0	0	0	1
III–IV Stage	0	0	0	0	0	0
IV Stage	14	9	77	3	3	18

**Figure 5:**
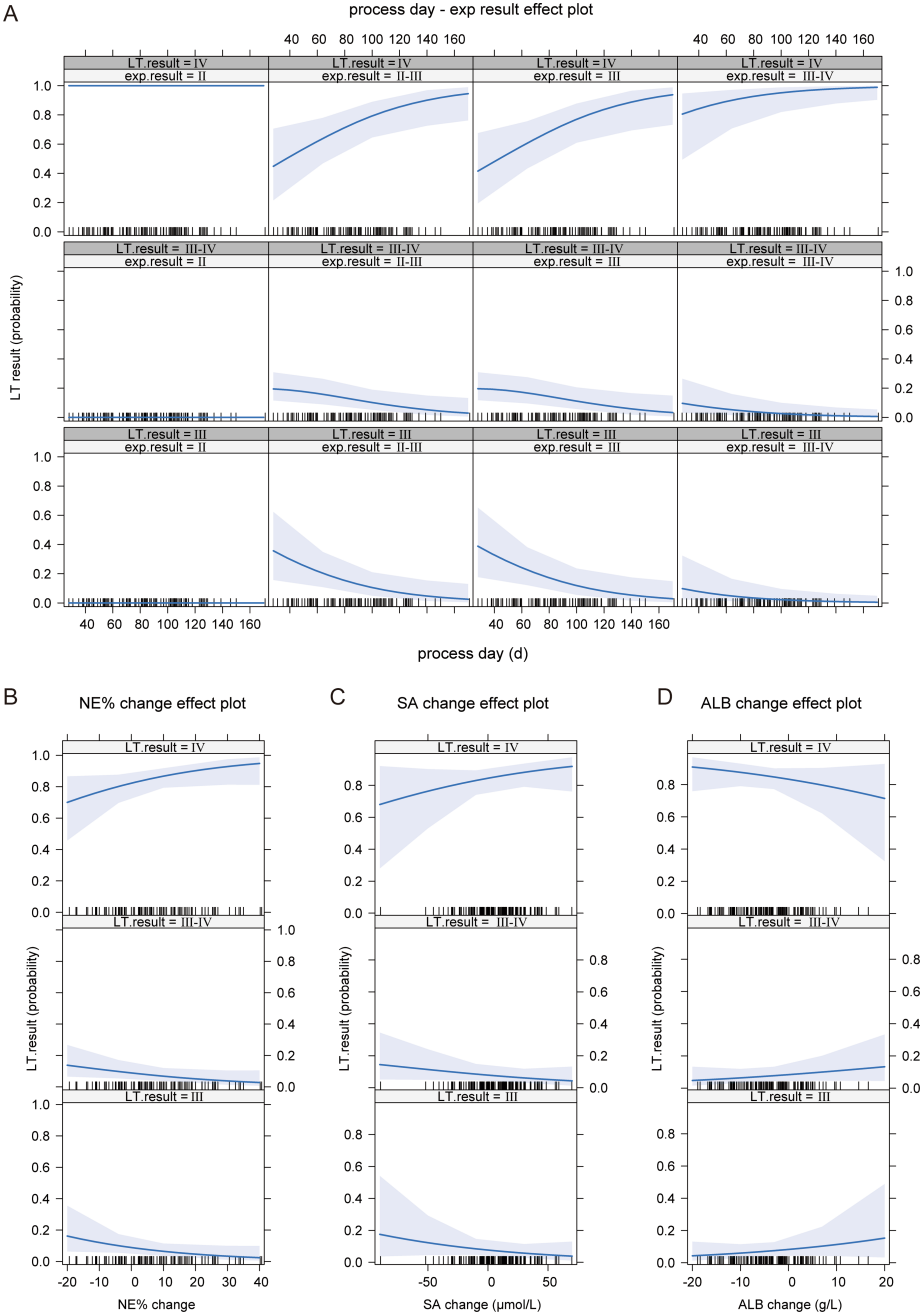
The impact of independent variables on the dependent variable in the ordinal logistic regression model. **(A)** The effect of BA liver cirrhosis staging at the time of examination and disease progression time on the progression of BA liver cirrhosis. **(B)** The impact of NE% change on the progression of BA liver cirrhosis. **(C)** The impact of SA change on the progression of BA liver cirrhosis. **(D)** The impact of ALB change on the progression of BA liver cirrhosis.

The model analysis also revealed that higher cirrhosis staging at the time of exploration and longer progression time were associated with more severe cirrhosis progression (see Figure 5A). Additionally, during the progression of BA, higher NE% and SA levels indicated more severe cirrhosis progression (Figures 5B,C). Although ALB levels increased at liver transplantation compared to exploration, a negative growth in ALB was indicative of more severe cirrhosis progression (Figure 5D).

## 4 Discussion

BA is a severe hepatobiliary disease primarily occurring in neonates, characterized by obstruction of both intrahepatic and extrahepatic bile ducts, which leads to impaired bile drainage and subsequent bile accumulation within the liver. This accumulation causes liver injury, liver cirrhosis, and eventually liver failure (1–3). Although BA is classified as a rare disease in China, based on the annual birth of nearly 10 million newborns and an incidence rate of approximately 1.06–1.8 cases per 10,000 live births in Asia (4, 5), it can be roughly estimated that over 1,000 new cases of BA are diagnosed in China each year. This results in a significant pressure on the prevention and treatment of the disease.

The key to treating BA in early identification, early diagnosis, and early surgical intervention to establish bile drainage. BA represents the most common indication for pediatric liver transplantation worldwide. While the Kasai portoenterostomy remains the primary initial intervention, its success is time-dependent, and many children still experience progressive fibrosis. Our study leveraged a unique clinical scenario—the waiting period for liver transplantation in children with a definitive BA diagnosis whose caregivers opted for primary transplant without Kasai portoenterostomy—to provide a rare window into the unmodified natural history of BA cirrhosis progression. This design allowed us to move beyond the well-documented static changes of liver failure and instead characterize the dynamic, rapid rate of change in clinical, biochemical, and pathological parameters over a defined period.

The most striking finding was the breathtaking speed of histologic progression. Within a mean interval of just 83 ± 28.37 days, the proportion of children with Stage IV cirrhosis surged from 14.4 to 77.1%, with over 62% of the entire cohort progressing to the most advanced stage. This observation underscored the extremely fast and irreversible nature of BA-driven fibrogenesis and provided a granular, time-anchored quantification of this progression. This has profound implications for clinical management, emphasizing the urgency of expediting transplantation and the critical need for strategies to slow fibrosis during the inevitable waiting period.

The average age of BA diagnosis at our center was 78.64 ± 31.43 days, and 22 children were already in Stage IV biliary cirrhosis at the time of diagnosis. Most children had missed the optimal window for Kasai portoenterostomy and had to undergo liver transplantation. This indicates that a significant number of pediatric surgeons, obstetricians, neonatologists, and pediatricians in primary hospitals still have insufficient knowledge of BA, leading to inadequate education for pregnant women and their families. As a result, many BA children do not receive timely and specialized evaluation and treatment. The stool color card is an economical and efficient screening tool that has been widely used in countries like Japan. It allows for earlier identification and surgery for BA children (11, 12). However, in China, this method is not yet widely practiced or popularized. To improve early detection and outcomes for BA, there is a need for broader education and the establishment of a comprehensive system for primary screening, diagnosis, referral, and treatment. This would help ensure that BA cases are identified and treated at the earliest possible stage, improving prognosis and reducing the burden on healthcare systems.

Although there are many studies on diagnostic biomarkers and models related to BA, such as MMP7, GGT, and total bile acids (13–15), these models, whether based on retrospective or prospective cohorts, typically use outcomes such as surgical exploration to confirm BA diagnosis or pathological results after Kasai portoenterostomy. They do not reflect the dynamic characteristics of BA progression. The reasons behind the rapid progression of BA-related liver cirrhosis in a short period remain unclear. However, this rapid progression may be one of the key factors contributing to the continuous advancement of liver cirrhosis after Kasai portoenterostomy, which ultimately leads to the need for liver transplantation. Therefore, in this study, we aimed to investigate the progression of BA-related liver cirrhosis in children at our center during the period between BA diagnosis and liver transplantation. By combining clinical laboratory parameters and liver pathological changes, we sought to explore the potential factors contributing to the progression of BA liver cirrhosis.

Therefore, this study primarily reviewed the laboratory examination data of children at two time points: prior to “intraoperative cholangiogram” and “liver transplantation” surgery preparation. The inclusion of laboratory examination data serves two main purposes: first, this study represents an initial exploration of the potential factors for BA liver cirrhosis; and second, given the current understanding of BA in grassroots hospitals, the aim is to identify easily accessible indicators that can predict the degree of cirrhosis progression and thus be easily promoted. The study found that indicators such as PLT, HB, LYM%, E%, ALB, PA, Cr, and LAC showed a decrease compared to the time of exploration. In contrast, WBC, NE, NE%, LYM, TBIL, DBIL, IBIL, AST, ALT, AST/ALT, TP, GLB, TBA, and SA all significantly increased compared to the time of exploration. However, no significant changes were observed in WBC, E, PBMC, and GGT between the two time points.

Further analysis of the indicators that undergo progressive changes during disease progression shows that most of the changes in blood components or biochemical data are secondary. For instance, the reduction in HB and Cr reflects that the children are in a state of liver dysfunction and malnutrition, often accompanied by coagulation disorders, esophageal varices, gastrointestinal bleeding, etc., leading to anemia and negative nitrogen balance. Similarly, the sustained increase in TBIL, DBIL, and IBIL, particularly the increase in DBIL, is due to bile duct obstruction, causing bile retention and progressively worsening jaundice in the patients. The continuous rise in transaminases (AST, ALT) and SA reflects the progressive decline in liver function. However, while some of these changes may be secondary, they could also be contributing factors to the ongoing progression of the disease. These factors should not be overlooked. Early and proactive intervention during or after the Kasai procedure, or while awaiting liver transplantation, may significantly improve the prognosis.

In the progression of BA, the increase in WBC, NE, LYM, and NE% suggests that the BA patients are in a state of systemic inflammation. These changes could be due to factors such as pneumonia in the patients or the physiological changes associated with the growth and development stages, particularly in NE and LYM. However, whether these changes might contribute to or accelerate the progression of liver cirrhosis remains an important consideration. Given that systemic inflammation can influence liver injury and fibrosis, it is essential to further explore how these inflammatory markers impact the overall progression of BA-related liver cirrhosis. Monitoring and managing inflammation in these patients could potentially offer therapeutic targets to slow the progression of liver damage. It is widely accepted that immune factors play a significant role in the pathogenesis and progression of BA, especially the involvement of lymphocytes, such as Th17 and Treg cells (2, 16). In the context of altered serum protein composition, despite impaired synthetic function in these patients, the increase in TP levels is intriguing. When TP is divided into its components—ALB and GLB—a decrease in ALB levels reflects impaired liver synthetic function, while an increase in GLB levels suggests potential activation of B lymphocytes, which could lead to the production of immunoglobulins. However, whether these immunoglobulins are functionally active remains uncertain. Compared to T lymphocytes, there has been relatively limited research on the role of B lymphocytes in BA. However, some studies have reported similar phenomena. For example, B cell infiltration has been observed in the residual bile duct tissue in BA patients, and these B cells produce oligoclonal Ig complexes (17). In the liver of BA patients, B lymphocytes have been found to generate autoreactive IgG antibodies due to defects in tolerance. Interestingly, depleting B lymphocytes has been shown to result in some degree of liver function improvement (16).

Total bile acid (TBA) levels were previously thought to increase as a secondary manifestation of impaired bile excretion in conditions like BA. However, recent studies have started to focus on the role of TBA in the pathogenesis and progression of BA. Research has categorized bile acids into different subtypes for use in diagnostic models for BA or predictive models for the prognosis of Kasai portoenterostomy (14, 18). These studies have found that TBA levels, along with conjugated bile acids (such as glucuronide-conjugated, taurine-conjugated bile acids) and deoxycholic acid, are significantly higher in BA patients compared to healthy controls. Even when symptoms of bile stasis are alleviated after Kasai portoenterostomy, the levels and composition of bile acids still show differences from healthy individuals. Interestingly, the use of inhibitors targeting the ileal bile acid transporter (IBAT), which regulates bile acid absorption in the small intestine, has been shown to delay liver remodeling in BA patients (18, 19). This suggests that bile acids, particularly those that are conjugated or altered in BA, might play a crucial role in liver damage and fibrosis progression. Modulating bile acid absorption could be a potential therapeutic strategy for managing liver fibrosis and stasis, even after surgical intervention. More studies are needed to further explore how bile acid subtypes contribute to BA and to validate therapeutic approaches targeting bile acid metabolism.

## 5 Conclusion

Our study has several limitations. First, its single-center, retrospective design inherits the associated biases. Second, our cohort represents a specific subset of BA patients, limiting the generalizability of our findings. Third, unmeasured confounding factors could have influenced some laboratory values. Finally, the use of mean imputation for missing data is a limitation. Future research should focus on external validation and elucidating the mechanistic drivers of the observed associations through integrated multi-omics approaches.

In conclusion, by analyzing the “waiting period” for transplantation, this study provides a unique longitudinal perspective on the natural history of BA progression. We confirm its alarming rapidity and illuminate the concomitant dynamics of immune activation. The predictive model we propose offers a potential framework for risk-stratifying children on the transplant waitlist. Ultimately, these findings reinforce the concept of BA as a dynamic inflammatory fibrotic disorder and highlight the urgent need for effective adjuvant therapies to slow disease progression and bridge children safely to transplantation.

## Data Availability

The original contributions presented in the study are included in the article/supplementary material, further inquiries can be directed to the corresponding authors.

## Glossary

ALB: Albumin
ALT: Alanine aminotransferase
AST: Aspartate aminotransferase
BA: Biliary atresia
Cr: Creatinine
DBIL: Direct bilirubin
E: Eosinophil
GGT: Gamma-glutamyltransferase
GLB: Globulin
HB: Hemoglobin
IBIL: Indirect bilirubin
LAC: Serum lactic acid
LT: Liver transplantation
LYM: Lymphocyte
NE: Neutrophil
PA: Prealbumin
PBMC: Peripheral blood mononuclear cell
PLT: Platelet
RBC: Red blood cell
SA: Serum ammonia
TBA: Total bile acids
TBIL: Total bilirubin
TP: Total protein
WBC: White blood cell

## References

[1] Hartley JL Davenport M Kelly DA. Biliary atresia. Lancet. (2009) 374:1704–13. doi: 10.1016/S0140-6736(09)60946-6, PMID: 19914515

[2] Bezerra JA Wells RG Mack CL Karpen SJ Hoofnagle JH Doo E. Biliary atresia: clinical and research challenges for the twenty-first century. Hepatology. (2018) 68:1163–73. doi: 10.1002/hep.29905, PMID: 29604222 PMC6167205

[3] Ortiz-Perez A Donnelly B Temple H Tiao G Bansal R Mohanty SK. Innate immunity and pathogenesis of biliary atresia. Front Immunol. (2020) 11:329. doi: 10.3389/fimmu.2020.00329, PMID: 32161597 PMC7052372

[4] Lee KJ Kim JW Moon JS Ko JS. Epidemiology of biliary atresia in Korea. J Korean Med Sci. (2017) 32:656–60. doi: 10.3346/jkms.2017.32.4.656, PMID: 28244293 PMC5334165

[5] Hsiao CH Chang MH Chen HL Lee HC Wu TC Lin CC. Universal screening for biliary atresia using an infant stool color card in Taiwan. Hepatology. (2008) 47:1233–40. doi: 10.1002/hep.22182 18306391

[6] Chardot C Carton M Spire-Bendelac N Le Pommelet C Golmard J-L Auvert B. Epidemiology of biliary atresia in France: a national study 1986–1996. J Hepatol. (1999) 31:1006–13. doi: 10.1016/S0168-8278(99)80312-2 10604573

[7] McKiernan PJ Baker AJ Kelly DA. The frequency and outcome of biliary atresia in the UK and Ireland. Lancet. (2000) 355:25–9. doi: 10.1016/S0140-6736(99)03492-3, PMID: 10615887

[8] Yoon PW Bresee JS Olney RS James LM Khoury MJ. Epidemiology of biliary atresia: a population-based study. Pediatrics. (1997) 99:376–82. doi: 10.1542/peds.99.3.376, PMID: 9041292

[9] Nemeh C Schmoke N Wu YS Wang P Lagana SM Remotti H. Clinical utility of intraoperative wedge biopsies after preoperative core needle biopsies in biliary atresia. Am J Surg. (2025) 245:116367. doi: 10.1016/j.amjsurg.2025.116367, PMID: 40319559

[10] Gao F Chen YQ Fang J Gu SL Li L Wang XY. Acoustic radiation force impulse imaging for assessing liver fibrosis preoperatively in infants with biliary atresia: comparison with liver fibrosis biopsy pathology. J Ultrasound Med. (2017) 36:1571–8. doi: 10.7863/ultra.16.08043, PMID: 28407283

[11] Tseng JJ Lai MS Lin MC Fu YC. Stool color card screening for biliary atresia. Pediatrics. (2011) 128:e1209–15. doi: 10.1542/peds.2010-3495 22025588

[12] Gu Y-H Yokoyama K Mizuta K Tsuchioka T Kudo T Sasaki H. Stool color card screening for early detection of biliary atresia and long-term native liver survival: a 19-year cohort study in Japan. J Pediatr. (2015) 166:897–902.e1. doi: 10.1016/j.jpeds.2014.12.063, PMID: 25681196

[13] Lertudomphonwanit C Mourya R Fei L Zhang Y Gutta S Yang L. Large-scale proteomics identifies MMP-7 as a sentinel of epithelial injury and of biliary atresia. Sci Transl Med. (2017) 9:eaan8462. doi: 10.1126/scitranslmed.aan8462, PMID: 29167395 PMC5902315

[14] Han YJ Hu SQ Zhu JH Cai X Lai DM Chen BH. Accurate prediction of biliary atresia with an integrated model using MMP-7 levels and bile acids. World J Pediatr. (2023) 20:822–33. doi: 10.1007/s12519-023-00779-7 38141111 PMC11402860

[15] Jiang J Wang J Shen Z Lu X Chen G Huang Y. Serum MMP-7 in the diagnosis of biliary atresia. Pediatrics. (2019) 144:e20190902. doi: 10.1542/peds.2019-0902, PMID: 31604829

[16] Wang J Xu Y Chen Z Liang J Lin Z Liang H. Liver immune profiling reveals pathogenesis and therapeutics for biliary atresia. Cell. (2020) 183:1867–1883.e26. doi: 10.1016/j.cell.2020.10.048, PMID: 33248023

[17] Taylor SA Malladi P Pan X Wechsler JB Hulse KE Perlman H. Oligoclonal immunoglobulin repertoire in biliary remnants of biliary atresia. Sci Rep. (2019) 9:4508. doi: 10.1038/s41598-019-41148-7, PMID: 30872727 PMC6418100

[18] Takeda M Takei H Suzuki M Tsukui T Tsuboi K Watayo H. Bile acid profiles in adult patients with biliary atresia who achieve native liver survival after portoenterostomy. Sci Rep. (2024) 14:2492. doi: 10.1038/s41598-024-52969-6, PMID: 38291117 PMC10827714

[19] Laue T Baumann U. Odevixibat: an investigational inhibitor of the ileal bile acid transporter (IBAT) for the treatment of biliary atresia. Expert Opin Investig Drugs. (2022) 31:1143–50. doi: 10.1080/13543784.2022.2151890, PMID: 36440482

